# Tree shape and bearing shoot type jointly drive within-canopy differentiation of fruit quality and mineral element allocation in jujube

**DOI:** 10.3389/fpls.2026.1869677

**Published:** 2026-07-20

**Authors:** Xijin Zhao, Xuanyu Liu, Zhenlei Wang, Renci Xiong, Minjuan Lin

**Affiliations:** 1 National-Local Joint Engineering Laboratory of High Efficiency and Superior-Quality Cultivation and Fruit Deep Processing Technology on Characteristic Fruit Trees/Technology Innovation Center for Characteristic Forest Fruits in Southern Xinjiang, Alar, China; 2 Corps Key Laboratory of Conservation and Utilization of Biological Resources in Tarim Basin, Alar, China; 3 College of Horticulture and Forestry, Tarim University, Alar, China

**Keywords:** jujube, tree shape, bearing shoot type, canopy structure, fruit quality, spatial heterogeneity

## Abstract

**Introduction:**

Tree shape optimizes canopy architecture; however, its role in regulating within-canopy spatial heterogeneity of fruit quality and the underlying physiological mechanisms remains unclear in jujube (*Ziziphus jujuba Mill.*). We hypothesized that tree shape drives fruit quality differentiation by restructuring canopy structure, thereby modulating bearing shoot type distribution and mineral element partitioning.

**Methods:**

A two-year (2024-2025) field trial was conducted on 15-year-old 'Junzao' trees. Following an initial screening of four tree shapes, the two superior forms—Small-canopy shape (S) and Trunk shape (T)—were selected for in-depth analysis in 2025. Canopies were vertically stratified into upper, middle, and lower layers, and fruits were classified by bearing shoot type (lignified vs. deciduous). Canopy structural parameters (LAI, MTA, DIFN), fruit quality traits (sugars, phenolics, flavonoids), mineral elements (K, Ca, Mg, etc.), and yield components were systematically evaluated.

**Results and Discussion:**

Optimized tree shapes (S and T) significantly outperformed Open-central shape and unpruned controls in overall fruit quality. Vertically, a distinct trade-off emerged: single-fruit weight and soluble sugars peaked on lignified shoots in the middle canopy (29.08 g, 26.66%), while total flavonoids and phenolics accumulated predominantly in the upper and lower layers. Potassium distribution aligned closely with sugar accumulation, whereas calcium and magnesium were enriched in zones with higher diffuse light penetration (linked to lower LAI and higher DIFN). Deciduous shoots accumulated more mineral elements than lignified shoots within the same layer, suggesting a compensatory uptake mechanism. Notably, the Small-canopy shape increased yield per plant by 31.46%, Grade 1 fruit percentage by 45.05%, and yield per unit area by 40.49% compared to the Trunk shape. PCA clearly separated the two shapes, with mineral elements (K, Ca, Mg) and quality traits (single-fruit weight, total phenolics) serving as key discriminators. Our results demonstrate that tree shape orchestrates within-canopy quality differentiation through structural modulation of canopy light environments and shoot-type composition, which subsequently alters mineral element source-sink partitioning. These findings provide a mechanistic basis for precision pruning strategies aimed at improving fruit uniformity and marketable yield in jujube production.

## 1 Introduction

The jujube (*Ziziphus jujuba* Mill.) is a distinctive fruit tree native to China that is gaining increasing global attention due to its unique nutritional value and economic importance (Liu et al., 2014). Among the many cultivated varieties, ‘Junzao’ stands out for its excellent horticultural traits—such as large fruit size, high yields, and rich nutritional value—and has become one of the primary varieties grown (Xu et al., 2023). As a result, market demand for high-quality ‘Junzao’ fruits continues to grow. Fruit quality encompasses appearance, substances affecting taste (such as sugar and acid), and health-promoting functional components (such as flavonoids and phenols), and ultimately determines its commercial value and competitiveness in the market (Wang C et al., 2022).

The overall quality of fruit is the result of complex interactions among genotype, environmental factors, and cultivation practices (Iwata et al., 2016; Bertin and Génard, 2018). Among the controllable cultivation factors, tree shape is considered one of the most effective artificial means of optimizing the microclimate within the canopy, photosynthetic efficiency, and the transport pathways of assimilates between “sources and sinks” (Posada et al., 2009). The tree shape created through pruning directly determines the spatial distribution of light within the canopy, ventilation conditions, and pathways for nutrient transport (Jiménez-Brenes et al., 2017). For example, studies on fruit trees such as apple (Da Silva et al., 2014) and pear (Zhang et al., 2025) have shown that tree shape directly determines branch architecture and leaf spatial arrangement, which in turn governs light interception, penetration, and within-canopy temperature gradients. By modifying these canopy structural parameters, optimized tree shapes such as the Trunk shape and Small-canopy shape can significantly improve light capture and light penetration within the canopy, thereby enhancing fruit yield and quality. However, for cultivars such as ‘Junzao’, research on the physiological mechanisms by which tree shape influences fruit quality remains relatively limited (Liu et al., 2020).

Although research on tree shapes has demonstrated their impact on overall fruit yield, most current studies still focus on comparing the overall average yield and quality differences between different tree shapes (Anthony and Minas, 2021; Dian et al., 2023). Such microenvironmental variations (e.g., light, temperature, and humidity) directly influence leaf photosynthesis, transpiration rate, and nutrient uptake efficiency, which collectively determine the quality of individual fruits. A key yet often overlooked issue is that, even within the same tree shape, due to significant vertical gradients in environmental factors such as light and temperature, the branch architecture, leaf physiology, and fruit development at different levels of the canopy may exhibit systematic “spatial heterogeneity” (Bailey, 2019; Jin et al., 2024; Zhang et al., 2025). These internal variations determine the uniformity of fruit production and the proportion of high-quality fruit on individual trees, and their importance is no less significant than that of differences in tree shape. More importantly, it remains unclear how canopy microenvironmental heterogeneity—driven by tree structure—regulates the distribution and transport of mineral elements across organs and different spatial locations within the canopy. This heterogeneity is characterized by vertical gradients in light intensity, temperature, and humidity. The mineral elements involved, such as K, Ca, and B, are closely linked to phloem loading, transpiration-driven xylem flow, and cell wall metabolism, thereby directly modulating sugar accumulation, fruit hardness, and overall quality. Thus, elucidating the mechanisms underlying the chain of “tree shape—microenvironment—distribution of mineral elements—fruit quality formation” is key to bridging horticultural practices (tree shaping) and fruit quality formation.

We hypothesized that tree shape drives within-canopy fruit quality differentiation through two interrelated mechanisms: (1) restructuring canopy architecture and the vertical distribution of bearing shoot types creates spatially distinct microenvironments; (2) these structural changes subsequently modulate the partitioning of mineral elements between source and sink organs, which in turn determines the spatial patterns of fruit quality. To test this hypothesis, we designed and implemented a two-year (2024-2025) field trial: in 2024, four tree shapes (Trunk shape, Small-canopy shape, Open-central shape, and CK) were screened for overall fruit quality; in 2025, the two superior shapes were vertically stratified into upper, middle, and lower layers to measure canopy structure, fruit quality, mineral elements, and yield. The findings are expected to provide tailored pruning strategies for jujube production based on within-canopy spatial heterogeneity, contributing to improved fruit uniformity, nutritional quality, and sustainable orchard management.

## 2 Material and methods

### 2.1 Plant materials

**Figure 1:**
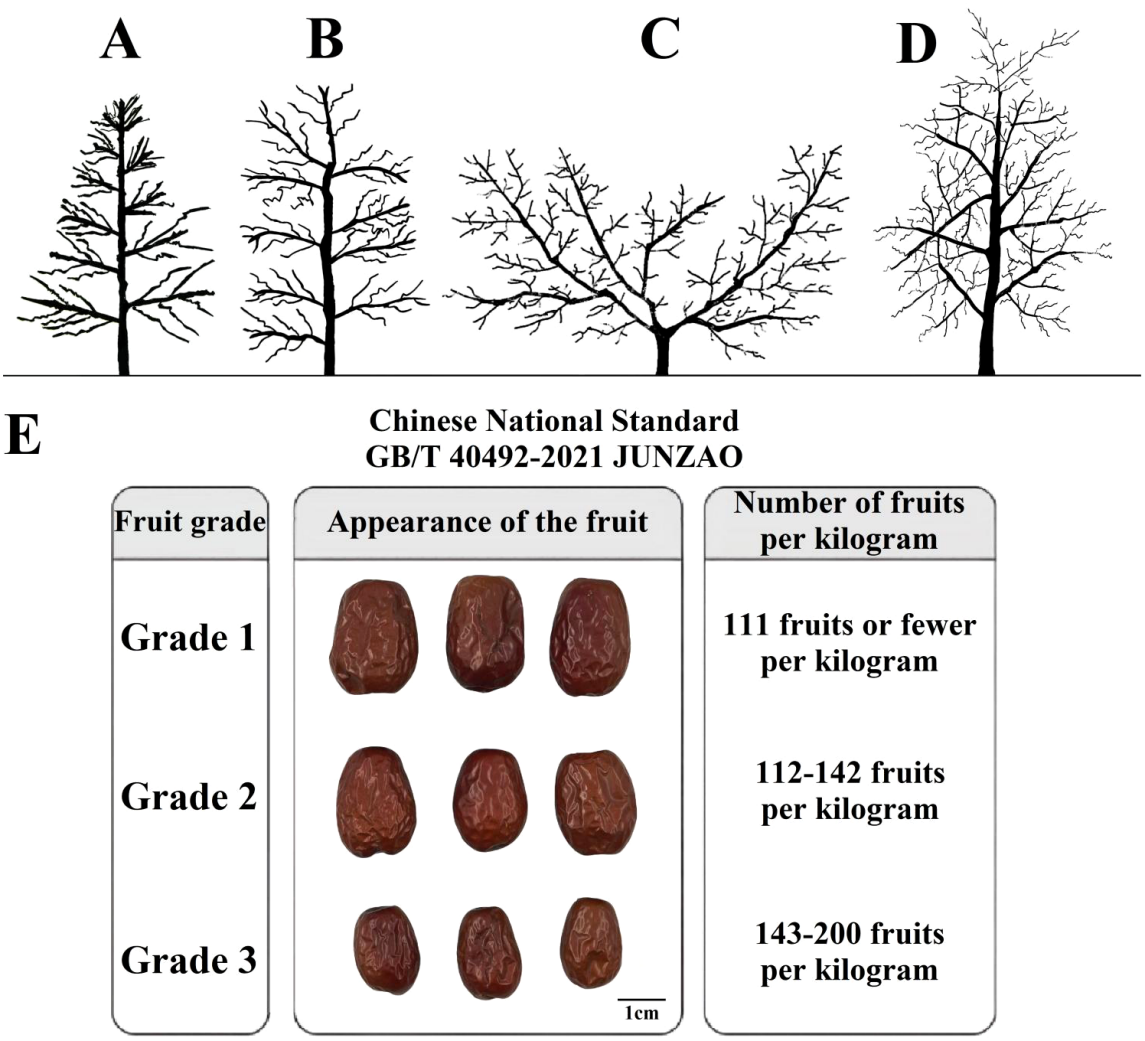
Different tree shapes of ‘Junzao’ jujube: **(A)** Small-canopy shape; **(B)** Trunk-shape; **(C)** Open-central shape; **(D)** CK; **(E)** Chinese national standard GB/T 40492-2021 “JUNZAO”.

We used ‘Wild jujube’ (*Ziziphus jujuba* var. *spinosa*) as the rootstock and selected 15-year-old ‘Junzao’ trees. From 2024 onward, trees were managed according to their respective shapes with consistent practices within each treatment. Small-canopy shape trees: The key to tree management lies in creating a clear, compact, and well-defined structural system. Key management practices include: selecting main branches that are well-spaced and do not obstruct one another, training them into different tiers while maintaining sufficient vertical spacing, and ensuring a clear hierarchical structure (central leader > primary branch > sub-lateral) (**Figure 1A**). Trunk-shape trees: This tree shape is designed to establish and maintain a dominant, vertical, centralized leadership structure. The primary management practice is to eliminate a highly competitive branch structure (**Figure 1B**). Open-central shape trees: The tree-shaped management approach aims to create an open canopy without a central leader. The primary pruning practice involves removing central leader shoots to maintain an open-central shape tree structure (**Figure 1C**). CK: Allow it to grow naturally without pruning (**Figure 1D**). They maintain the same levels of watering and fertilization. Row spacing is 2 × 4 m. All trees were irrigated via drip irrigation with canal water derived from melting snow and glaciers of the Tianshan Mountains, which is the predominant water source in the southern Xinjiang region of China. We irrigate five times a year at the following approximate dates corresponding to key phenological stages: late March-early April (before bud break), mid-May (before flowering), early July (first fruit enlargement), early August (second fruit enlargement), and late October-early November (before soil freezing), using 200 cubic meters of water per hectare each time. Water-soluble compound fertilizer (Shikefeng Chemical Industry Co.Ltd.) is applied three times a year via drip fertigation at a rate of 50 kg/ha per application, coinciding with the irrigations before bud break, before flowering, and during early fruit enlargement. Total nutrient content of the fertilizer ≥40%, with a N-P_2_O_5_-K_2_O ratio of 2:1:1. In this study, 100 healthy trees per tree shape (400 trees in total) free of pests and diseases (with similar heights and consistent growth patterns) were randomly selected based on four growth forms: trunk-shaped trees, small-canopy trees, open-central trees, and CK trees.

Fruit sampling was conducted in both 2024 and 2025 in late September (September 20-25) during the fully red stage, defined as fully red peel with no softening (Yan et al., 2022). The same maturity criterion was applied in both years to ensure comparability; prior to formal sampling, 10 fruits per tree were randomly collected every 3 days from September 10 to monitor peel color and flesh firmness, and formal sampling commenced once all monitored fruits met the fully red standard. In each year, five trees were randomly selected from each tree shape, and 60 fruits were collected per tree from the middle to upper portion of bearing shoots, with 15 fruits taken from each of the four cardinal directions (east, south, west, and north). For the 2024 sampling, fruits were collected without canopy stratification for the analysis of six quality parameters: single-fruit weight, soluble sugar content, titratable acidity, soluble protein content, total flavonoid content, and total phenolic content. For the 2025 sampling, fruits were additionally stratified into three canopy layers (upper, middle, and lower), which were divided into three equal vertical sections between the lowest and highest fruiting branches on each tree. Within each layer, 20 fruits were collected (5 fruits per cardinal direction) for the analysis of the same six quality parameters plus mineral element content (P, K, Fe, Ca, Mg, and B). After single-fruit weight measurement, all fruits from each sample were pitted, chopped, mixed, and stored at -20 °C until analysis, which was completed within two weeks after each sampling (i.e., by early October of each year).

### 2.2 Test methods

#### 2.2.1 Determination of tree parameters

In October 2025, researchers measured the tree parameters of the upper, middle, and lower canopies of the selected tree species. According to previous reports (Liu et al., 2014; Zhang et al., 2025), three trees were measured for each parameter of each tree shape. Each tree serves as a replicate sample in the experiment. We carefully counted the number of mother bearing shoot, bearing shoot, second shoot, fruits, lignified bearing shoot, and deciduous bearing shoot.

#### 2.2.2 Determination of canopy structures

In June 2025, the LAI-2200c canopy analyzer (LI-COR, USA) was used to measure canopy parameters for plants in each treatment, including leaf area index (LAI), mean tilt angle (MTA), and diffuse non-interceptance (DIFN). For each tree shape, five trees were randomly selected from each canopy layer, and a 14-meter-long crisscross survey transect was laid out between rows so that the transect coverage would accurately reflect the overall structural characteristics of the canopy. Each path was measured three times, with the instrument calibrated and white balance adjusted before each measurement. During the operation, the operator should stand away from the probe to avoid obstructing the optical reading.

#### 2.2.3 Determination of the fruit quality parameters of jujube fruit

Individual fruit weight was measured using an FA 1104 N electronic balance (Shanghai Pohai Instrument Company, Shanghai, China). The longitudinal and transverse diameters of the fruit were measured using an electronic vernier caliper (Shanghai Deyixing Tools Co., LTD, Shanghai, China). The soluble solids content was determined using a PAL-1 digital handheld refractometer (ATAGO Co., LTD, Tokyo, Japan). For each tree, 30 fruits were measured as subsamples; the mean value of these 30 measurements was then calculated to represent that individual tree, and the tree-level means (n = 3 per tree shape) were used as biological replicates for statistical analysis.

Determination of soluble sugar content using the anthrone colorimetric method (Kuang et al., 2017). Briefly, 0.1 g of freeze-dried fruit powder was extracted with 10 mL distilled water at 80 °C for 30 min, and the supernatant was collected after centrifugation. The supernatant was mixed with anthrone reagent (0.2% anthrone in 98% sulfuric acid) and heated in a boiling water bath for 10 min. After cooling to room temperature, the absorbance was measured at 620 nm, and the concentration was calculated using a glucose standard curve.

Determination of titratable acid content by acid-base neutralization titration (Pu et al., 2018): 5.0 g of fresh pulp was homogenized with 50 mL distilled water, filtered, and the filtrate was titrated with 0.1 M NaOH to pH 8.1; the result was expressed as the percentage of citric acid equivalents.

The soluble protein content was determined using the Coomassie Blue G-250 staining method (Snyder et al., 1978): 0.5 g of fresh pulp was ground in phosphate buffer (pH 7.0), centrifuged, and the supernatant was reacted with Coomassie G-250 reagent; absorbance was read at 595 nm against a bovine serum albumin (BSA) standard curve.

Total flavonoid was determined by the aluminum colorimetric method (Duan et al., 2023): 1.0 g of dried powder was extracted with 70% ethanol, and the extract was sequentially reacted with 5% NaNO_2_, 10% AlCl_3_·6H_2_O, and 1 M NaOH; the absorbance at 510 nm was measured, and rutin was used as the standard.

Total phenols was determined by the Folin-Ciocalteu method (Casajús et al., 2021): the extract was mixed with Folin-Ciocalteu reagent and 7.5% Na_2_CO_3_, incubated in the dark, and absorbance was recorded at 765 nm, with gallic acid as the standard. All samples were analyzed in triplicate for each of the above parameters.

#### 2.2.4 Determination of jujube fruit yield

In October 2025, 40 trees of each shape were evaluated to record the fruit yield (total fresh fruit weight) of each individual tree. Each tree serves as a replicate sample. According to the Chinese national standard GB/T 40492-2021 “JUNZAO”, ‘Junzao’ fruits are graded based on the number of fruits per kilogram (Grade 1: 111 fruits or fewer per kilogram). The fruit must be large and plump, exhibit the characteristic traits of the ‘Junzao’ variety, have red to purplish-red skin, thick flesh, and be free of rot or mold. Based on the above criteria, the processed fruits are sorted and tallied. The formula for calculating the percentage of Grade 1 fruits is: Percentage of Grade 1 fruits = (Number of Grade 1 fruits/Total number of fruits) × 100%.

At the same time, we monitored the 5-hectare experimental site. Yield per unit area (t/ha) was calculated by summing the fruit weight of all trees in each hectare of the experimental plot. Each hectare of experimental plot serves as a replicate for the experiment.

#### 2.2.5 Determination of mineral element content in jujube fruits

Fruit samples (50 g) were dried at 60 °C for 72 hours, ground to a fine powder, and passed through a 200-mesh sieve. The resulting powder (0.5 g) was subjected to microwave digestion system (Nanjing Kejie Testing Technology Development Co., Ltd., Nanjing, China) using a mixture of HNO_3_ and H_2_O_2_ (5:1, v/v). The mineral element content, including P, K, Fe, Ca, Mg, and B, was quantified using inductively coupled plasma optical emission spectrometer (ICP-OES) (Beijing Leibotech Instruments Co., Ltd., Beijing, China). The ICP-OES was operated under the following conditions: RF power of 1300 W and an atomizer flow rate of 0.7 L/min. The concentrations of the target elements were determined based on the recorded sample mass and digest volume, following a previously established method. To ensure analytical quality, the following quality control measures were implemented: a certified plant reference material (GBW07603, National Institute of Metrology, China) was digested and analyzed with each batch of samples to verify accuracy; reagent blanks were prepared and analyzed for background correction. All mineral element concentrations are expressed on a dry weight basis. Each sample was analyzed in three technical replicate.

### 2.3 Data processing and analysis

Data processing and analysis were performed using IBM SPSS Statistics version 26.0 (IBM, Armonk, NY, USA). Data were first tested for normality (Shapiro-Wilk test) and homogeneity of variances (Levene’s test) prior to parametric analysis. Duncan’s multiple range test was then applied as a *post-hoc* procedure to compare means within each significant main effect or interaction, following the protected ANOVA principle (i.e., *post-hoc* tests were performed only when the ANOVA F-test indicated significant differences). The significance level was set to *p* < 0.05. For key parameters, we also performed Bonferroni-adjusted pairwise comparisons as a sensitivity check; the significance patterns were consistent with those obtained from Duncan’s test. The correlation analyses and mapping were conducted with Chiplot (https://www.chiplot.online/) (URL accessed on 9 February 2026). Principal component analyses and plotting were performed using Origin 2021 (Origin Lab Inc., Northampton, MA, USA).

## 3 Results

### 3.1 Fruit quality analysis of different tree shapes of jujube trees

**Figure 2:**
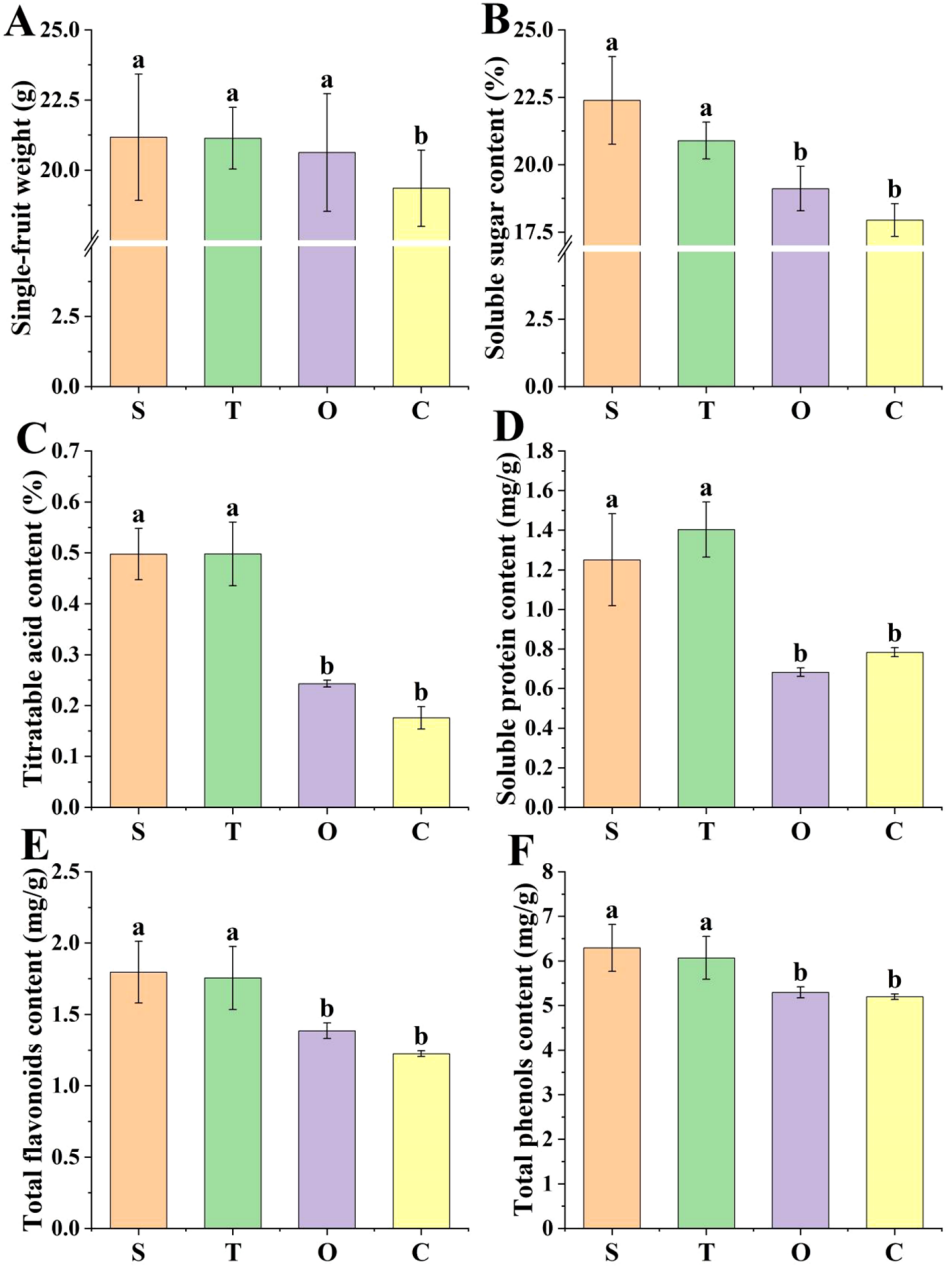
Fruit quality of different tree shapes of jujube trees. **(A)** single-fruit weight; **(B)** soluble sugar content; **(C)** titratable acid content; **(D)** soluble protein content; **(E)** total flavonoid content; **(F)** total phenolic content. S, Small-canopy shape; T, Trunk shape; O, Open-central shape; C, ck. Different lowercase letters in the figures indicate significant differences (*p* < 0.05).

Fruit quality varied significantly among the four tree shapes (**Figure 2**). Compared with Open-central shape and CK, the Small-canopy shape and Trunk shape exhibited 9.32%-24.74% higher soluble sugar content, 104.90%-183.60% higher titratable acid content, and 59.51%-105.52% higher soluble protein content (all *p* < 0.05). CK had the lowest single-fruit weight (19.35 g), which was 8.60% lower than the Small-canopy shape (21.17 g, *p* < 0.05). On the other hand, the total flavonoid (26.66%-46.62%) and total phenolic contents (14.62%-21.09%) of fruits with the Small-canopy shape and Trunk shape were significantly higher than those of fruits with the Open-central shape and ck. These results indicate that the Small-canopy shape and Trunk shape have a clear advantage in maintaining nutrient accumulation in the fruit. Therefore, the Small-canopy shape and Trunk shape were selected as the subjects for further mechanistic analysis.

### 3.2 Analysis of tree parameters and canopy structure of different tree shapes and layers of jujube trees

**Figure 3:**
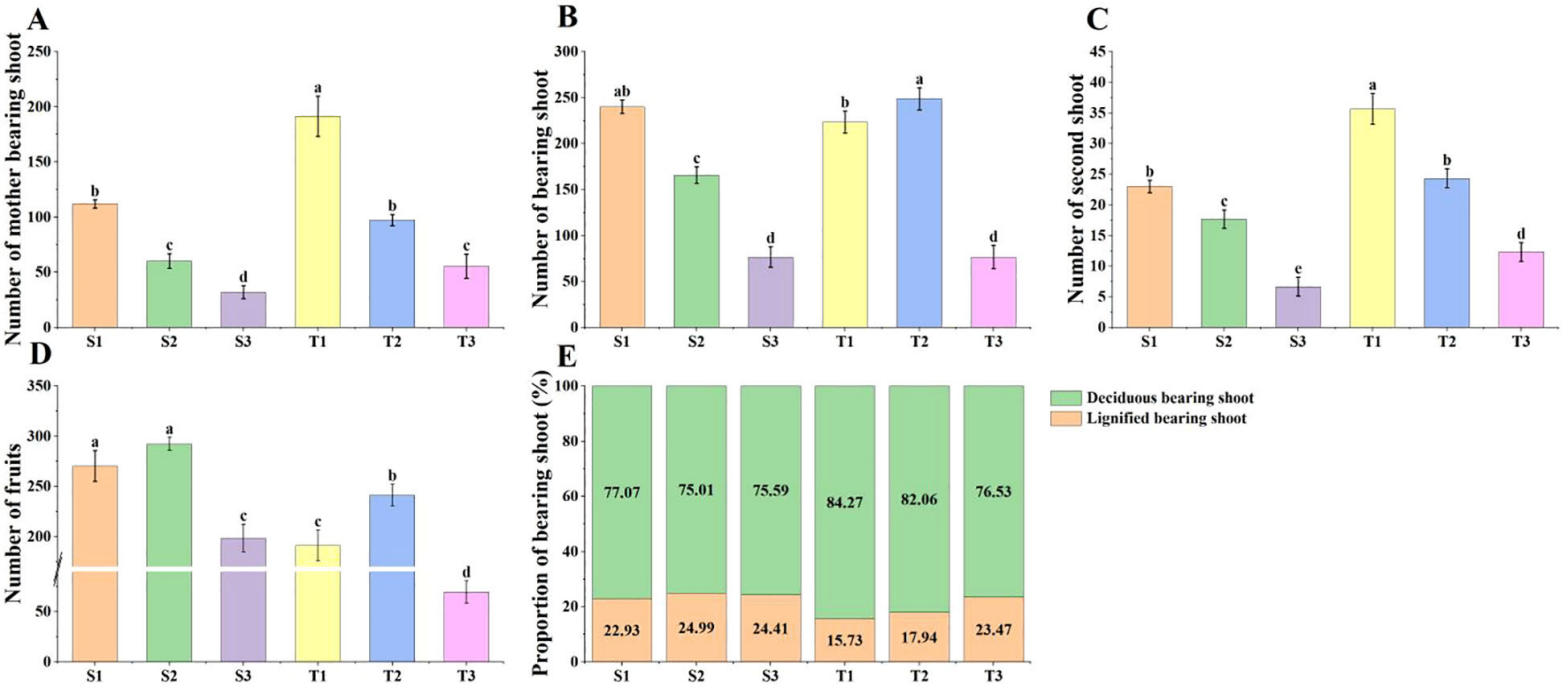
Tree parameters of different tree shapes and layers of jujube trees. **(A)** number of mother bearing shoot; **(B)** number of bearing shoot; **(C)** number of second shoot; **(D)** number of fruits; **(E)** proportion of bearing shoot. S, Small-canopy shape; T, Trunk shape. The numbers 1, 2 and 3 in the figure respectively represent the upper, middle and lower layers of the tree, and the same applies below. Different lowercase letters in the figures indicate significant differences (*p* < 0.05).

**Figure 4:**
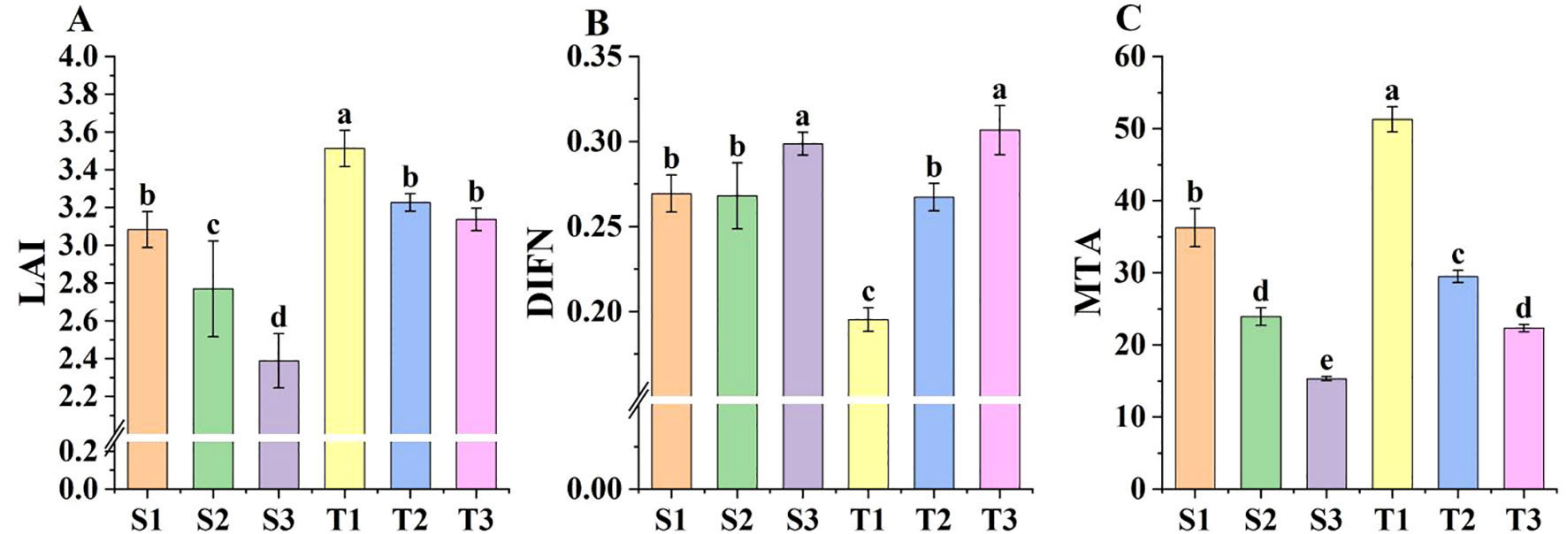
Canopy structure of different tree shapes and layers of jujube trees. **(A)** leaf area index (LAI); **(B)** diffuse non-interceptance (DIFN); **(C)** mean tilt angle (MTA). Different lowercase letters in the figures indicate significant differences (*p* < 0.05).

Tree parameters and canopy structure showed marked vertical heterogeneity within both tree shapes (**Figures 3**, **4**). In the Trunk shape, the upper layer (T1) had 46.58%-243.71% more mother bearing shoots and secondary shoots than T2 and T3. In the Small-canopy shape, fruit number was highest in the middle and upper layers (S1+S2), which together bore 73.94% of total fruits per tree. Across all layers, deciduous bearing shoots dominated (75.01%-84.27%), but the lignified shoot proportion was highest in S2 (24.99%) and lowest in T1 (15.73%).

Canopy structure parameters corroborated these vertical patterns: LAI and MTA in T1 were higher than in lower layers, while DIFN (a proxy for light penetration) was 1.11- to 1.57-fold greater in the lower layers (S3, T3) than in the upper layers (all *p* < 0.05). These structural gradients indicate that both tree shapes create vertically stratified light environments that likely influence resource allocation.

### 3.3 Analysis of fruit quality of different tree shapes and layers of jujube trees

Primary metabolites (single-fruit weight and soluble sugars): These two parameters displayed highly concordant spatial patterns (**Table 1**). Single-fruit weight was higher on lignified shoots (L) than on deciduous shoots (D) across all layers and both shapes, with the maximum recorded in S2L (29.08 g) and the minimum in T3D (17.28 g). Soluble sugar content similarly peaked in S2L and S3L (26.66% and 26.64%, respectively).

**Table 1:** Fruit quality of different tree shapes and layers of jujube trees.

Sample type	Single-fruit weight (g)	Soluble sugar content (%)	Titratable acid content (%)	Soluble protein content (mg/g)	Total flavonoid content (mg/g)	Total phenols content (mg/g)
S1L	17.64 ± 0.48 e	21.53 ± 2.13 c	0.46 ± 0.08 ab	7.70 ± 0.22 a	1.71 ± 0.11 cd	7.82 ± 0.20 ab
S1D	17.72 ± 1.26 e	22.28 ± 1.14 c	0.38 ± 0.08 bc	7.94 ± 0.19 a	1.69 ± 0.11 cd	8.08 ± 0.23 a
S2L	29.08 ± 0.80 a	26.66 ± 0.71 a	0.42 ± 0.06 bc	7.80 ± 0.35 a	1.67 ± 0.08 cd	7.25 ± 0.24 d
S2D	18.37 ± 1.17 e	24.42 ± 2.22 b	0.35 ± 0.08 bc	7.57 ± 0.33 a	2.27 ± 0.36 b	6.95 ± 0.16 e
S3L	25.26 ± 1.92 b	26.64 ± 3.03 a	0.47 ± 0.07 a	7.80 ± 0.34 a	1.72 ± 0.03 cd	8.16 ± 0.10 a
S3D	18.30 ± 1.90 e	23.98 ± 0.05 b	0.45 ± 0.03 ab	7.89 ± 0.86 a	1.78 ± 0.06 cd	6.98 ± 0.25 e
T1L	21.97 ± 0.97 c	26.09 ± 1.20 a	0.39 ± 0.08 bc	7.58 ± 0.31 a	1.61 ± 0.03 d	6.67 ± 0.36 f
T1D	18.37 ± 1.47 e	24.46 ± 1.53 b	0.33 ± 0.10 c	7.95 ± 0.18 a	1.95 ± 0.11 c	6.65 ± 0.16 f
T2L	21.41 ± 0.92 c	25.36 ± 0.86 ab	0.30 ± 0.05 c	7.87 ± 0.14 a	1.80 ± 0.28 cd	6.80 ± 0.73 ef
T2D	19.89 ± 0.90 d	26.68 ± 1.66 a	0.42 ± 0.04 bc	7.91 ± 0.31 a	1.69 ± 0.10 cd	7.67 ± 0.32 b
T3L	21.16 ± 0.87 c	22.03 ± 1.34 c	0.38 ± 0.05 bc	8.18 ± 0.31 a	2.86 ± 0.18 a	7.48 ± 0.15 c
T3D	17.28 ± 1.96 e	22.92 ± 1.20 bc	0.38 ± 0.07 bc	7.91 ± 0.31 a	1.89 ± 0.14 cd	7.66 ± 0.12 b

Different lowercase letters in the table indicate significant differences (*p* < 0.05).

Secondary metabolites (total flavonoids and total phenolics): In striking contrast to sugars, bioactive compounds accumulated most heavily in the upper and lower layers (**Table 1**). Total flavonoids peaked in T3L (2.86 mg/g) and were 71.26%-77.64% higher than the lowest values (S2L and T1L, 1.61-1.67 mg/g). Total phenolics ranged from 6.65 mg/g (T1D) to 8.16 mg/g (S3L), with the Small-canopy shape showing 17.24%-21.50% higher phenolics than the Trunk shape in the upper layers.

Titratable acidity varied narrowly (0.30%-0.47%), with S3L slightly higher than T2L (*p* < 0.05). Soluble protein content was unaffected by tree shape, canopy layer, or shoot type (range: 7.57-8.18 mg/g), indicating constitutive stability of this parameter.

### 3.4 Analysis of mineral element contents in fruits of different tree shapes and layers of jujube trees

Mineral element concentrations were strongly influenced by tree shape, canopy layer, and shoot type, with distinct accumulation patterns for each element (**Table 2**). K and Ca—the two most abundant elements—showed contrasting spatial distributions. K was enriched in the lower canopy of both shapes, with the highest value in T3D (11518.68 mg/kg) and the lowest in T1L (3030.52 mg/kg)—a 3.80-fold difference. In the Small-canopy shape, Ca peaked in the middle layer (S2D: 4726.36 mg/kg), which was 2.82-fold higher than T1L, whereas Ca in the Trunk shape remained uniformly low across all layers.

**Table 2:** Mineral element contents in fruits of different tree shapes and layers of jujube trees.

Sample type	P (mg/kg)	K (mg/kg)	Fe (mg/kg)	Ca (mg/kg)	Mg (mg/kg)	B (mg/kg)
S1L	341.34 ± 6.45 h	6647.94 ± 105.01 g	18.60 ± 0.97 gh	1982.31 ± 20.24 g	360.99 ± 5.29 g	57.34 ± 1.23 i
S1D	491.56 ± 12.74 e	8711.42 ± 850.77 de	87.61 ± 2.41 a	2872.74 ± 22.57 f	622.82 ± 4.48 d	68.56 ± 1.30 h
S2L	432.64 ± 13.50 g	9398.92 ± 457.98 c	55.04 ± 4.61 b	3957.15 ± 70.87 b	677.11 ± 14.23 b	112.29 ± 4.94 e
S2D	466.73 ± 5.41 f	8886.76 ± 117.90 cd	38.05 ± 2.58 ef	4726.36 ± 10.93 a	702.65 ± 7.73 a	138.04 ± 4.10 d
S3L	508.43 ± 17.53 de	10956.55 ± 235.40 a	34.89 ± 0.97 f	3588.73 ± 64.68 cd	540.19 ± 4.89 e	181.64 ± 2.45 b
S3D	514.44 ± 4.92 d	10212.19 ± 312.34 b	20.39 ± 0.62 g	3530.74 ± 27.45 d	629.56 ± 15.20 d	204.15 ± 2.37 a
T1L	318.98 ± 7.85 i	3030.52 ± 100.07 i	16.27 ± 0.97 h	1235.95 ± 4.11 i	291.79 ± 3.25 h	49.75 ± 1.06 j
T1D	493.87 ± 4.04 e	4979.81 ± 172.35 h	16.45 ± 0.81 gh	1534.61 ± 10.78 h	432.41 ± 7.71 f	74.49 ± 2.36 g
T2L	499.37 ± 10.71 de	5036.85 ± 61.75 h	43.78 ± 2.06 d	1962.39 ± 51.28 g	358.01 ± 8.62 g	91.42 ± 0.67 f
T2D	860.99 ± 12.10 a	7511.38 ± 311.84 f	41.53 ± 2.73 de	2876.89 ± 59.62 f	547.13 ± 1.01 e	175.12 ± 4.88 c
T3L	631.85 ± 8.77 c	8204.00± 141.55 e	49.36 ± 2.96 c	2967.93 ± 14.29 e	549.19 ± 0.24 e	77.26 ± 1.73 g
T3D	840.42 ± 16.00 b	11518.68 ± 443.95 a	39.72 ± 1.33 e	3631.29 ± 64.79 c	652.46 ± 12.39 c	88.13 ± 5.43 f

Different lowercase letters in the table indicate significant differences (*p* < 0.05).

P, Mg, B, and Fe also exhibited element-specific vertical patterns. P peaked in T2D and T3D (860.99 and 840.42 mg/kg), while Mg was highest in S2D and S2L (702.65 and 677.11 mg/kg). B showed a striking accumulation in S3D (204.15 mg/kg)—4.10-fold higher than T1L (49.75 mg/kg)—and Fe was exceptionally high in S1D (87.61 mg/kg), exceeding all other samples by 0.59- to 4.38-fold.

Among the two tree shapes, deciduous bearing shoots (D) on the same layer generally accumulated higher levels of mineral elements than lignified bearing shoots (L). This compensatory enrichment in structurally weaker shoots suggests an adaptive mechanism to enhance nutrient acquisition under competitive disadvantage.

### 3.5 Yield analysis of different tree shapes and layers of jujube trees

**Figure 5:**
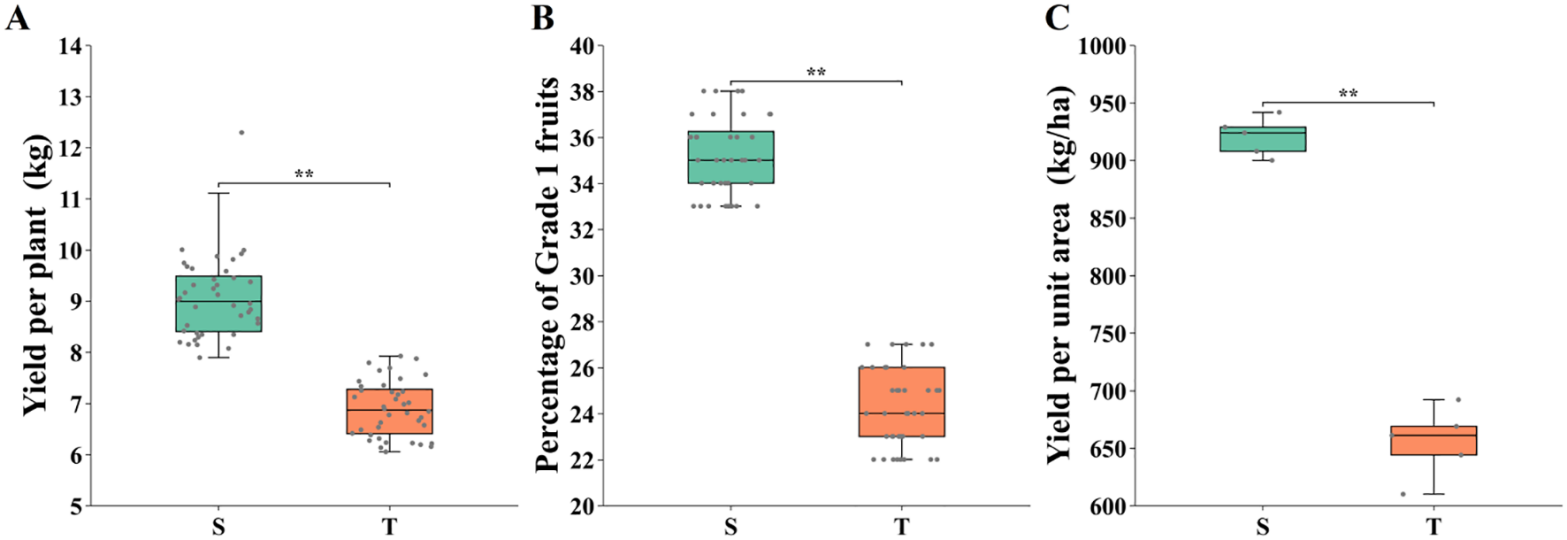
Yield of different tree shapes of jujube. **(A)** yield per plant; **(B)** percentage of Grade 1 fruits; **(C)** yield per unit area. S, Small-canopy shape; T, Trunk shape. * indicates a significant difference (*p* < 0.05) and ** indicates a highly significant difference (*p* < 0.01).

Yield performance significantly favored the Small-canopy shape over the Trunk shape (**Figure 5**). Specifically, the Small-canopy shape achieved 31.46% higher yield per plant, 45.05% more Grade 1 fruits, and 40.49% greater yield per unit area compared to the Trunk shape (all *p* < 0.05).

### 3.6 Correlation analysis of different tree shapes jujube trees

**Figure 6:**
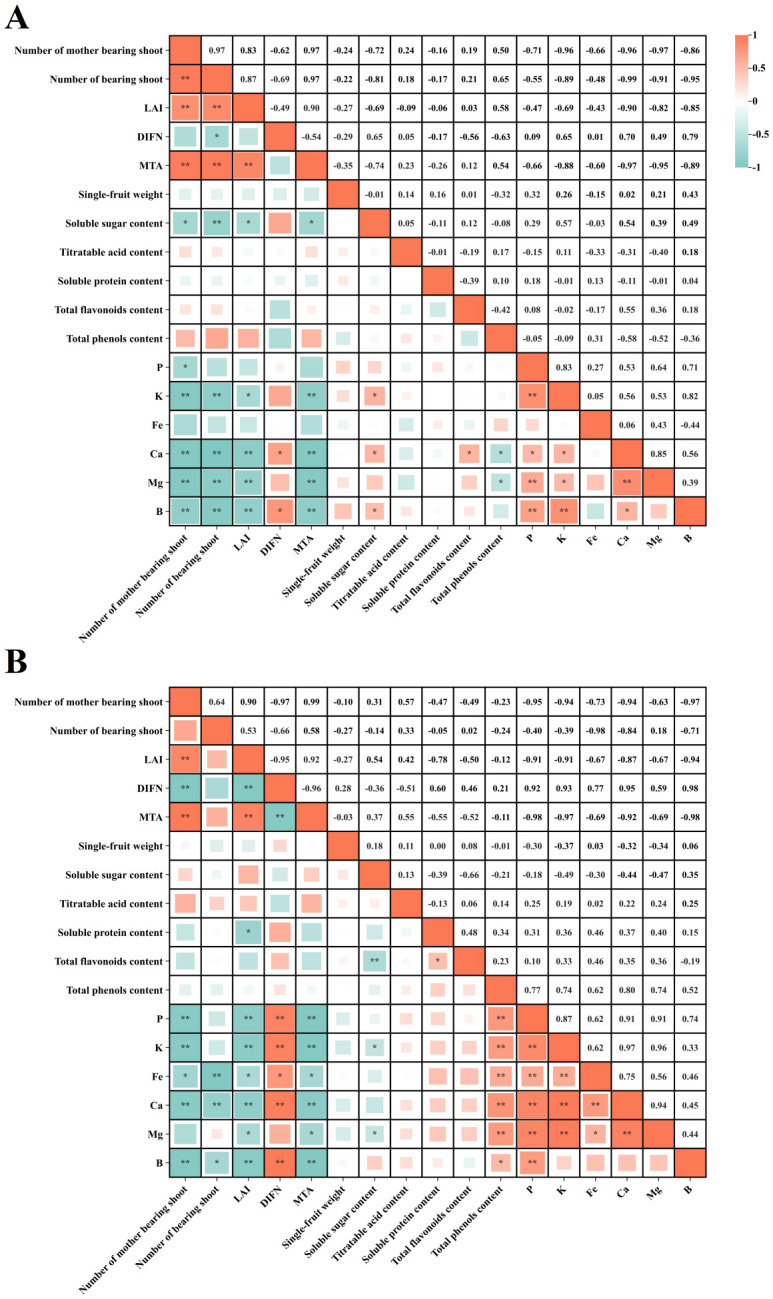
Correlation analysis of different tree shapes jujube trees. **(A)** small-canopy shape; **(b)** trunk shape. * indicates a significant difference and ** indicates a highly significant difference.

Correlation analyses revealed how canopy structure mediates the relationships between mineral elements and fruit quality, with distinct patterns between the two tree shapes (**Figure 6**). In the Small-canopy shape, K, Ca, and B were all significantly and positively correlated with soluble sugar content (r = 0.49-0.57, *p* < 0.05), while Ca and Mg showed significant negative correlations with total phenolics (r = –0.52 to –0.58). These results suggest that K/Ca/B promote sugar accumulation, whereas Ca/Mg may suppress phenolic biosynthesis. Additionally, LAI and MTA were strongly negatively correlated with K, Mg, and soluble sugars, indicating that dense upper canopies restrict both mineral transport and sugar loading.

In the Trunk shape, K and Mg were negatively correlated with soluble sugars (r = –0.47 to –0.49, *p* < 0.05)—a pattern opposite to that observed in the Small-canopy shape. P and Fe were negatively associated with LAI and MTA but positively correlated with DIFN, suggesting that light penetration enhances P and Fe accumulation in lower canopy layers. These divergent correlation patterns between the two shapes highlight that tree architecture fundamentally alters the physiological coupling between mineral nutrition and fruit quality formation.

### 3.7 Principal component analysis of different tree shapes jujube trees

**Figure 7:**
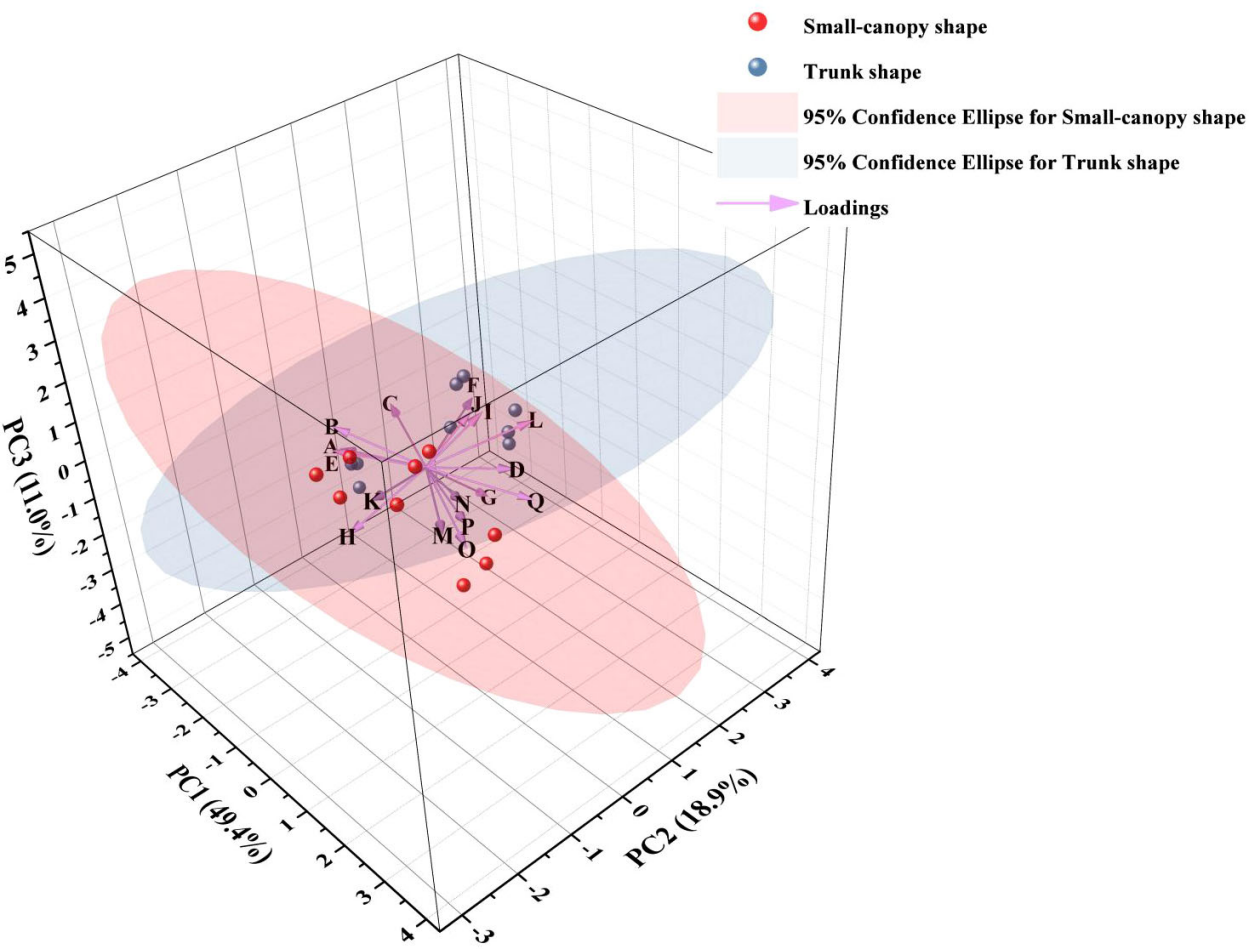
Principal component analysis of different tree shapes jujube trees. **(A)** Number of mother bearing shoot; **(B)** Number of bearing shoot; **(C)** LAI; **(D)** DIFN; **(E)** MTA; **(F)** Single-fruit weight; **(G)** Soluble sugar content; **(H)** Titratable acid content; **(I)** Soluble protein content; **(J)** Total flavonoids content; **(K)** Total phenols content; **(L)** phosphorus (P); **(M)** potassium (K); **(N)** iron (Fe); **(O)** calcium (Ca); **(P)** magnesium (Mg); **(Q)** boron (B).

Principal component analysis (PCA) was performed on all standardized variables, including tree parameters, canopy structure indicators, fruit quality traits, and mineral element concentrations (**Figure 7**; **Table 3**). The first three principal components explained 49.40%, 18.90%, and 11.00% of the total variance, respectively, with a cumulative contribution of 79.30%.

**Table 3:** Loadings of each principal component.

Code in Figure 7	Indicators	PC1	PC2	PC3
A	Number of mother bearing shoot	-0.3255	-0.1191	-0.0670
B	Number of bearing shoot	-0.2454	-0.1981	0.2945
C	Leaf area index (LAI)	-0.3146	0.1259	0.1078
D	Diffuse non-interceptance (DIFN)	0.2697	0.1710	0.1116
E	Mean tilt angle (MTA)	-0.3248	-0.1557	-0.0471
F	Single-fruit weight	-0.1899	0.4147	0.0106
G	Soluble sugar content	-0.0059	0.2859	-0.5723
H	Titratable acid content	0.0065	-0.3815	-0.1858
I	Soluble protein content	0.1187	0.1786	0.4335
J	Total flavonoids content	-0.0063	0.2400	0.2504
K	Total phenols content	0.1512	-0.3679	0.2770
L	Phosphorus (P)	0.2056	0.3353	0.2992
M	Potassium (K)	0.3109	-0.2175	0.0121
N	Iron (Fe)	0.2689	-0.0832	0.0840
O	Calcium (Ca)	0.3181	-0.1257	-0.2008
P	Magnesium (Mg)	0.3075	-0.1046	-0.0404
Q	Boron (B)	0.2766	0.2445	-0.2332

PC1 (49.40%)—the “mineral–structure factor”: This component was strongly and positively loaded by K (0.3109), Ca (0.3181), and Mg (0.3075), and negatively loaded by the number of mother bearing shoots (–0.3255), MTA (–0.3248), and LAI (–0.3146) (**Table 3**). Thus, PC1 primarily separates samples with high mineral element accumulation (positive side) from those with dense, structurally complex canopies (negative side). This suggests a trade-off between vegetative canopy development and mineral nutrient enrichment in fruits.

PC2 (18.90%)—the “quality trade-off factor”: This component was positively loaded by single-fruit weight (0.4147) and soluble sugar content (0.2859), but negatively loaded by total phenolics (–0.3679) and titratable acid content (–0.3815) (**Table 3**). PC2 therefore captures the well-known “high sugar vs. high phenolics” trade-off observed in our vertical layer analysis, separating fruits that prioritize primary metabolite accumulation (positive) from those that accumulate more secondary metabolites (negative).

PC3 (11.00%)—the “metabolic regulation factor”: This component was positively loaded by soluble protein (0.4335) and total flavonoids (0.2504), but negatively loaded by soluble sugars (–0.5723) (**Table 3**). PC3 further distinguishes samples based on the balance between different metabolic pathways, particularly separating sugar-rich fruits from those with higher protein and flavonoid contents.

Critically, the two tree shapes were clearly separated along PC1 and PC2 (**Figure 7**): 66.67% of the Small-canopy shape samples fell into the PC1^+^/PC2^+^ quadrant (high mineral elements + high single-fruit weight/sugars), whereas 66.67% of the Trunk-shape samples fell into the PC1^–^/PC2^–^ quadrant (dense canopy structure + high phenolics). This separation demonstrates that the Small-canopy shape simultaneously promotes mineral accumulation and primary metabolite synthesis, while the Trunk shape favors secondary metabolite accumulation under a denser canopy structure. These distinct coordination patterns between mineral nutrition and fruit quality traits further confirm that tree shape fundamentally restructures the physiological coupling within the canopy.

## 4 Discussion

### 4.1 Tree shapes reshape canopy structure and create vertical heterogeneity

Both optimized tree shapes exhibited significant vertical heterogeneity in bearing shoot composition and canopy structure (**Figures 3**, **4**), a pattern often overlooked when comparing only average differences between tree shapes (Saudreau et al., 2011; Da Silva et al., 2014).

The physiological significance of this heterogeneity lies in its impact on source-sink relationships. In the Trunk shape, the upper canopy (T1) developed a dense bearing shoot system (46.58%-243.71% more mother and secondary shoots than lower layers), providing an extensive “source” structure for assimilate production. However, this dense architecture simultaneously reduced light penetration to lower layers (DIFN increased 1.11- to 1.57-fold from upper to lower canopy). Such vertical gradients in light availability and branch density are known to modulate photosynthetic efficiency and assimilate partitioning (Da Silva et al., 2014; Yang et al., 2023), and our findings confirm that this general principle applies to jujube.

Importantly, across both tree shapes, deciduous bearing shoots (D) dominated numerically (75.01%-84.27% of all bearing shoots), yet lignified bearing shoots (L)—which possess more developed vascular tissue and lower hydraulic resistance—had higher fruit set and quality (Fan et al., 2017; Yang et al., 2021). This discrepancy highlights that evaluating productive capacity requires examining shoot type composition, not just total shoot number. The proportion of lignified shoots varied vertically: highest in S2 (24.99%) and lowest in T1 (15.73%), suggesting that tree shape regulates the vertical distribution of shoot types as part of resource allocation strategies (Kunz et al., 2019; Wang J et al., 2022).

The canopy structural gradients we observed—high LAI and MTA in upper layers versus increased DIFN in lower layers—are consistent with findings in apple (Da Silva et al., 2014) and pear (Zhang et al., 2025), confirming that this pattern extends to jujube. More importantly, by linking canopy structure parameters with shoot-type composition, our study establishes a hierarchical framework connecting architectural traits to resource allocation across vertical space.

Compared with previous studies, this research advances tree-shape analysis in two ways: shifting from “between-shape” to “within-shape” vertical comparisons, revealing non-uniform internal resource distribution even within optimized shapes; and incorporating shoot type as a microstructural unit, showing that the proportion of lignified vs. deciduous shoots varies systematically with canopy height (Bailey, 2019). These advances provide a structural foundation for understanding nutrient and assimilate partitioning across canopy strata.

### 4.2 Canopy structure and bearing shoot type jointly drive spatial differentiation in fruit quality

Fruit quality traits showed clear vertical differentiation patterns (**Table 1**), jointly driven by canopy position and bearing shoot type, confirming that resource distribution within jujube canopies is systematically structured rather than random (Losciale et al., 2010).

Primary metabolites (single-fruit weight and soluble sugars) consistently peaked on lignified bearing shoots in the middle canopy (S2L), exceeding deciduous bearing shoots in the same layer. This pattern reflects two interacting mechanisms. First, the middle canopy avoids both the photoinhibitory stress of the upper canopy (high light but potential temperature/UV extremes) and the severe shading of the lower canopy, providing a favorable light microenvironment for photosynthesis and fruit development (Lloyd et al., 2010). Second, lignified bearing shoots—with more developed xylem and phloem tissue—have lower hydraulic resistance and greater assimilate transport capacity, giving them a competitive advantage as “sink” organs in source-sink dynamics (Yang et al., 2021; Zhao et al., 2025). This supports the principle that sink strength, determined by both structural development and ecological position, governs assimilate partitioning within a plant (Bertin and Génard, 2018).

In the Trunk shape, high sugar content occurred in both T1L (upper, dense canopy) and T2D (middle, deciduous shoots), indicating that canopy position can partially compensate for shoot-type disadvantage—even structurally weaker shoots can be effective sinks when located in favorable microenvironments (He et al., 2013; Wang M et al., 2022). This interaction between shoot type and canopy position underscores the importance of considering both factors in quality management.

By contrast, bioactive compounds (flavonoids and phenolics) showed an opposite spatial distribution: highest in upper and lower canopy layers where sugar content was lowest—a clear “high sugar, low phenolics” vs. “low sugar, high phenolics” trade-off (**Table 1**). This pattern indicates carbon allocation competition between primary and secondary metabolism. In canopy zones with low LAI but high DIFN (e.g., T3, S3), the relatively open yet light-limited microenvironment likely restricts photosynthetic carbon supply, triggering a stress response that redirects available carbon toward the phenylpropanoid pathway for flavonoid and phenolic synthesis (Zoratti et al., 2014). Deciduous shoots (S2D) accumulated more flavonoids than lignified shoots in the same layer, further supporting that weaker sink strength (i.e., greater resource competition) promotes secondary metabolite accumulation as an adaptive strategy.

Titratable acid content varied narrowly (0.30%-0.47%), with only S3L slightly higher than other treatments, likely due to reduced nighttime respiration (and thus lower organic acid catabolism) in the cooler, shaded lower canopy (Zhang et al., 2025). Soluble protein remained stable across all treatments (7.57-8.18 mg/g), indicating constitutive homeostasis for this parameter.

### 4.3 Tree shapes drive the “source-sink” redistribution of mineral elements

Mineral element accumulation in fruits varied significantly across tree shapes, canopy layers, and shoot types (**Table 2**), with each element following a distinct spatial pattern governed by its specific transport physiology (Hocking et al., 2016; Nistor et al., 2022).

K distribution closely paralleled sugar accumulation patterns (**Table 1** and **Table 2**). K is essential for phloem loading: it maintains osmotic potential in sieve tubes and facilitates sucrose transmembrane transport in companion cells, particularly for photosynthates produced in high-light upper-canopy leaves (Rogiers et al., 2017; Ho et al., 2020; Luo et al., 2020). In both tree shapes, the highest K levels occurred in lower-canopy fruits (T3D: 11518.68 mg/kg; S3L: 10956.55 mg/kg), where sugars were also elevated, confirming that K availability supports sugar accumulation.

Ca exhibited an entirely different K. Unlike K, Ca is transported primarily via xylem transpiration and is immobile once deposited in fruit tissues (Kalcsits et al., 2020). Accordingly, Ca accumulation was highest in the middle canopy of the Small-canopy shape (S2D: 4726.36 mg/kg), where moderate LAI allowed sufficient light penetration and transpiration-driven flow, and lowest in the dense upper canopy of the Trunk shape (T1L: 1235.95 mg/kg), where high LAI reduces light penetration and internal humidity suppresses transpiration (Doyle et al., 2021). Correlation analysis confirmed Ca’s positive association with DIFN and negative association with LAI and MTA (**Figure 6**). As a key cell-wall component, adequate Ca supply is critical for fruit firmness and senescence delay (Huang et al., 2023).

In most cases, deciduous bearing shoots (D) accumulated significantly more mineral elements within the same canopy layer than lignified bearing shoots (L) (**Table 2**). This apparent paradox—structurally weaker shoots accumulating more nutrients—reflects a compensatory acquisition strategy. Deciduous shoots have less developed vascular tissue, reducing their competitive ability as sinks for phloem-mobile assimilates. To compensate, fruits on these shoots may enhance ion uptake and transport to ensure adequate mineral nutrition for basic physiological functions (Khan et al., 2023).

Limitations of this study should be acknowledged. First, although we initially screened four tree shapes, the in-depth mechanistic analysis focused exclusively on the two superior shapes (Small-canopy shape and Trunk shape). This focused approach enabled detailed vertical stratification and physiological characterization, but it limits direct comparison with other tree architectures (e.g., Open-central shape) and may not capture the full spectrum of physiological responses across all possible training systems. Second, the experiment was conducted at a single location in southern Xinjiang, China, under specific climatic, edaphic, and management conditions (drip irrigation, canal water from Tianshan snowmelt, 2 × 4 m spacing, and 15-year-old trees on wild jujube rootstock). While these conditions are representative of the major jujube-producing region in southern Xinjiang, the generalizability of our findings to other production areas—with different climates, soil types, irrigation regimes, or tree ages—remains to be tested. Future studies conducted across multiple locations and with a broader range of tree shapes would help validate the mechanistic framework proposed here and determine the extent to which the observed canopy structure–mineral element–fruit quality relationships are universally applicable or context-dependent. Despite these limitations, the consistency of our findings with established physiological principles (e.g., source-sink dynamics, transpiration-driven mineral transport, carbon allocation trade-offs) supports their broader relevance.

## 5 Conclusions

This study elucidates how tree shape regulates fruit quality in ‘Junzao’ jujube through a “canopy structure—mineral element distribution—quality formation” framework. Vertical heterogeneity in canopy structure and bearing shoot composition created spatially distinct microenvironments, driving a carbon allocation trade-off: sugars accumulated on lignified shoots in the middle canopy, while phenolics/flavonoids concentrated in upper and lower layers. Tree shape also redistributed mineral elements via distinct transport mechanisms—K with sugars via phloem loading, Ca/Mg via transpiration-driven xylem flow—with deciduous shoots showing compensatory mineral uptake. The Small-canopy shape outperformed Trunk shape in yield (31.46% higher) and Grade 1 fruit percentage (45.05% higher). For precision management, pruning should address within-canopy heterogeneity through thinning dense upper zones and optimizing shoot type ratios to improve fruit uniformity and quality.

## Data Availability

The original contributions presented in the study are included in the article/supplementary material. Further inquiries can be directed to the corresponding author.
